# Physiological Pattern of Disease Assessed by Pressure-Wire Pullback Has an Influence on Fractional Flow Reserve/Instantaneous Wave-Free Ratio Discordance: Insights From the Multicenter AJIP Registry

**DOI:** 10.1161/CIRCINTERVENTIONS.118.007494

**Published:** 2019-05-14

**Authors:** Takayuki Warisawa, Christopher M. Cook, James P. Howard, Yousif Ahmad, Shunichi Doi, Masafumi Nakayama, Sonoka Goto, Yohei Yakuta, Kenichi Karube, Matthew J. Shun-Shin, Ricardo Petraco, Sayan Sen, Sukhjinder Nijjer, Rasha Al Lamee, Yuki Ishibashi, Hisao Matsuda, Javier Escaned, Carlo di Mario, Darrel P. Francis, Yoshihiro J. Akashi, Justin E. Davies

**Affiliations:** 1 International Center for Circulatory Health, National Heart and Lung Institute, Imperial College London, Hammersmith Hospital, United Kingdom (T.W., C.M.C., J.P.H., Y.A., M.J.S.-S., R.P., S.S., S.N., R.A.L., D.P.F., J.E.D.).; 2 Division of Cardiology, Department of Internal Medicine, St. Marianna University School of Medicine, Kawasaki, Japan (T.W., Y.I., Y.J.A.).; 3 Department of Cardiovascular Medicine, St. Marianna University School of Medicine, Yokohama City Seibu Hospital, Japan (S.D., H.M.).; 4 Cardiovascular Center, Toda Central General Hospital, Japan (M.N., S.G.).; 5 Tokyo Women’s Medical University–Waseda University Joint Institution for Advanced Biomedical Sciences, Japan (M.N.).; 6 Cardiovascular Institute, Hospital Clínico San Carlos, Madrid, Spain (S.G., J.E.).; 7 Department of Cardiology, Kanazawa Cardiovascular Hospital, Japan (Y.Y.).; 8 Department of Cardiovascular Medicine, Okaya City Hospital, Japan (K.K.).; 9 Structural Interventional Cardiology, Careggi University Hospital, Florence, Italy (C.d.M.).

**Keywords:** coronary artery disease, fractional flow reserve, myocardial, hemodynamics, humans, registries

## Abstract

**Background:**

Fractional flow reserve (FFR) and instantaneous wave-free ratio (iFR) disagree on the hemodynamic significance of a coronary lesion in ≈20% of cases. It is unknown whether the physiological pattern of disease is an influencing factor for this. This study assessed whether the physiological pattern of coronary artery disease influences discordance between FFR and iFR measurement.

**Methods and Results:**

Three-hundred and sixty intermediate coronary lesions (345 patients; mean age, 64.4±10.3 years; 76% men) with combined FFR, iFR, and iFR pressure-wire pullback were included for analysis from an international multicenter registry. Cut points for hemodynamic significance were FFR ≤0.80 and iFR ≤0.89, respectively. Lesions were classified into FFR+/iFR+ (n=154; 42.7%), FFR−/iFR+ (n=38; 10.6%), FFR+/iFR− (n=41; 11.4%), and FFR−/iFR− (n=127; 35.3%) groups. The physiological pattern of disease was classified according to the iFR pullback recordings as predominantly physiologically focal (n=171; 47.5%) or predominantly physiologically diffuse (n=189; 52.5%). Median FFR and iFR were 0.80 (interquartile range, 0.75–0.85) and 0.89 (interquartile range, 0.86–0.92), respectively. FFR disagreed with iFR in 22% (79 of 360). The physiological pattern of disease was the only influencing factor relating to FFR/iFR discordance: predominantly physiologically focal was significantly associated with FFR+/iFR− (58.5% [24 of 41]), and predominantly physiologically diffuse was significantly associated with FFR−/iFR+ (81.6% [31 of 38]; *P*<0.001 for pattern of disease between FFR+/iFR− and FFR−/iFR+ groups).

**Conclusions:**

The physiological pattern of coronary artery disease was an important influencing factor for FFR/iFR discordance.

## Abstract 2

Supplemental Digital Content is available in the text.

**WHAT IS KNOWN**

- Proximal lesion location, diabetes mellitus, female sex, and differences in hyperemic coronary flow have all been suggested as factors contributing to fractional flow reserve (FFR)/instantaneous wave-free ratio (iFR) discordance.

**WHAT THE STUDY ADDS**

- The present study further demonstrated that the physiological pattern of coronary artery disease, as assessed by iFR pressure-wire pullback, also appeared a critical determinant of FFR/iFR discordance. Specifically, FFR+/iFR− discordance was more associated with physiologically focal disease, whereas FFR−/iFR+ discordance was more associated with physiologically diffuse disease. The performance of pressure-wire pullback as part of routine physiological stenosis assessment may provide useful additive information in cases of FFR/iFR discordance.

Coronary physiology permits vessel-specific identification of ischemia, and its use is recommended to guide revascularization decision-making in intermediate-severity coronary stenosis.^1^ The 2 most commonly used pressure-based indices in clinical practice are the fractional flow reserve (FFR) and the instantaneous wave-free ratio (iFR). FFR is measured during maximal hyperemia induced by vasodilating agents,^2^ whereas iFR is measured under resting conditions, without pharmacological hyperemia.^3^

It is well known that FFR and iFR disagree on the hemodynamic significance of a coronary lesion in ≈20% of cases.^4^ Previous studies showed influencing factors for FFR/iFR discordance included the lesion location,^5,6^ particular patient characteristics,^7^ and difference in hyperemic coronary flow.^8^ However, it is unknown whether the physiological pattern of coronary artery disease also influences FFR/iFR discordance.

In this study, we sought to determine whether the physiological pattern of coronary artery disease as assessed by iFR pullback was an influencing factor for FFR/iFR discordance.

## Methods

The data, analytic methods, and study materials will not be made available to other researchers for purposes of reproducing the results or replicating the procedure.

### Study Population

AJIP is an international multicenter registry that includes patients with intermediate coronary lesions and measurement of iFR pullback performed for clinical indications. This ongoing registry includes retrospective cases from the first-in-man iFR pullback case in March 9, 2015, and also will cover prospective cases by December 2018. From this registry, all consecutive cases (March 2015 to April 2018) with a combined measurement of FFR were included for this analysis (Figure I in the Data Supplement). Exclusion criteria were lesion severity >90% by visual estimation, acute coronary syndrome, previous coronary artery bypass grafting to the target vessel, vessels with angiographically identifiable myocardial bridging or collaterals, cardiac hypertrophy, and severe valvular pathology. All patients provided written informed consent. This study was approved by the local ethical committees at each participating center and was conducted according to the principles of the Declaration of Helsinki.

### Baseline Characteristics Data

Baseline characteristics data of the patients included age, sex, hypertension (defined as systolic

blood pressure ≥140 mm Hg, diastolic blood pressure ≥90 mm Hg, or the use of antihypertensive medication), dyslipidemia (defined as low-density lipoprotein cholesterol ≥140 mg/dL or the use of antilipidemic medication), diabetes mellitus (defined as hemoglobin A1c ≥6.5% or the use of antidiabetic medication), renal insufficiency (defined as estimated glomerular filtration rate <60 mL/min per 1.73 m^2^), and severe left ventricular systolic dysfunction (defined as left ventricular ejection fraction <30%).

### Coronary Catheterization and Measurement of Physiological Indices

Coronary angiography was performed using standard techniques according to routine clinical practice in the participating centers. The decision to perform FFR and iFR assessment was clinically oriented and thus left to operator’s discretion. Intracoronary nitrates (100–300 mg) were administered in all cases.

After normalization of the pressure wire (PrimeWire Prestige/Verrata; Philips, Amsterdam, the Netherlands) at the ostium of each vessel, the wire was advanced to the target vessel. FFR was calculated as the ratio of mean distal coronary pressure to mean aortic pressure across the whole cardiac cycle during maximal hyperemia. Hyperemia was induced by intravenous adenosine infusion (140–150 μg/kg per min; 334 of 360), intracoronary adenosine injection (40–150 μg; 24 of 360), or intracoronary nicorandil injection (2 mg; 2 of 360) as recommended.^9^ iFR was calculated as the ratio of mean distal coronary pressure to mean aortic pressure during the wave-free period of diastole.

Routine cutoff values of hemodynamic significance (FFR ≤0.80 and iFR ≤0.89) were used to classify stenoses into 4 groups: FFR+/iFR+ (FFR ≤0.80 and iFR ≤0.89), FFR−/iFR+ (FFR >0.80 and iFR ≤0.89), FFR+/iFR− (FFR ≤0.80 and iFR >0.89), and FFR−/iFR− (FFR >0.80 and iFR >0.89).

### iFR Pressure-Wire Pullback Measurement and Physiological Classification

iFR pullback recordings were performed manually at a pullback speed of ≈0.5 to 1.0 mm/s. After the pressure sensor reached the ostium of left main or right coronary artery as appropriate, the presence of pressure-wire drift was checked. If significant drift was identified, defined as ±0.02 units,^10^ measurements were repeated. Cases were excluded from the analysis if pressure-wire pullback was not performed from an adequately distal point of the vessel or if fluoroscopy was not provided to confirm the distal wire position.

**Figure 1.**
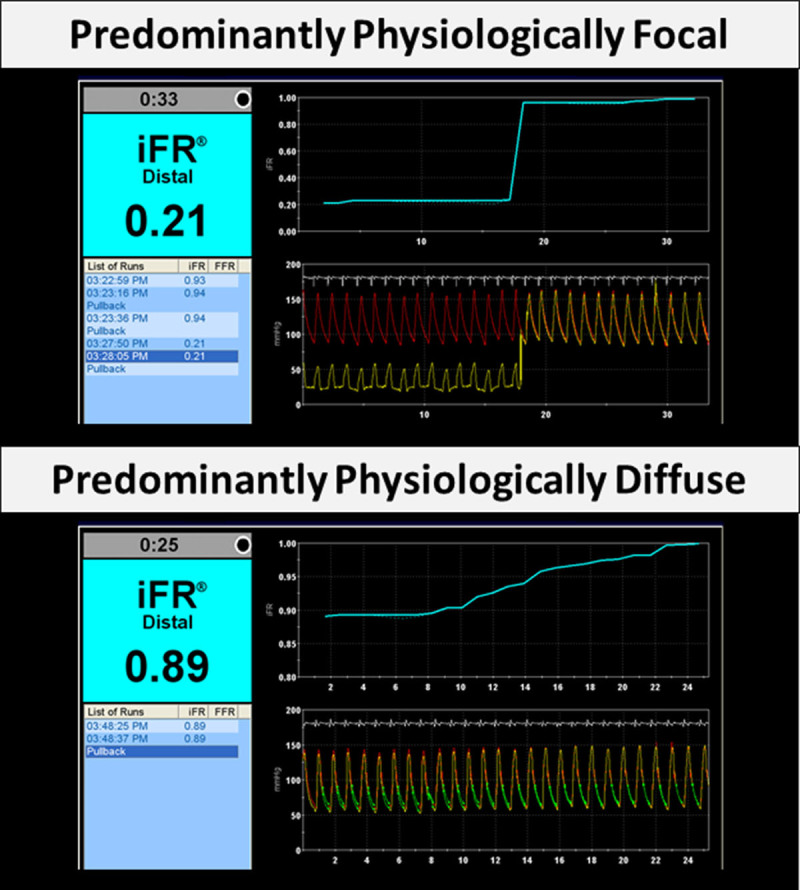
**Representative examples of different physiological patterns of coronary artery disease.** Predominantly physiologically focal disease (**top**) and predominantly physiologically diffuse disease (**bottom**). iFR indicates instantaneous wave-free ratio.

All pullback traces were jointly evaluated by 3 expert interventional cardiologists (R.P., S.S., and R.A.L.) who were blinded to the clinical presentation, patient characteristics, and coronary angiography. For each case, the consensus opinion for the physiological pattern of disease was generated by the unanimous agreement of the 3 experts. The physiological pattern of disease was classified as predominantly physiologically focal or predominantly physiologically diffuse by the consensus opinion. Representative examples of these classifications are shown in Figure 1.

### Statistical Analysis

Categorical data were expressed as numbers and percentages, whereas continuous variables were expressed as mean and (±) SD or as median accompanied by interquartile range as appropriate. Tests of normality were first performed using the Shapiro-Wilk test. Continuous variables were compared with Student *t* or Mann-Whitney *U* tests, and categorical variables with χ^2^ or Fisher exact tests, as appropriate. All probability values were 2 sided, and *P*<0.05 was considered statistically significant. All the statistical analysis was performed using R, version 3.2.1 (R Foundation for Statistical Computing, Vienna, Austria).

## Results

### Study Population

**Figure 2.**
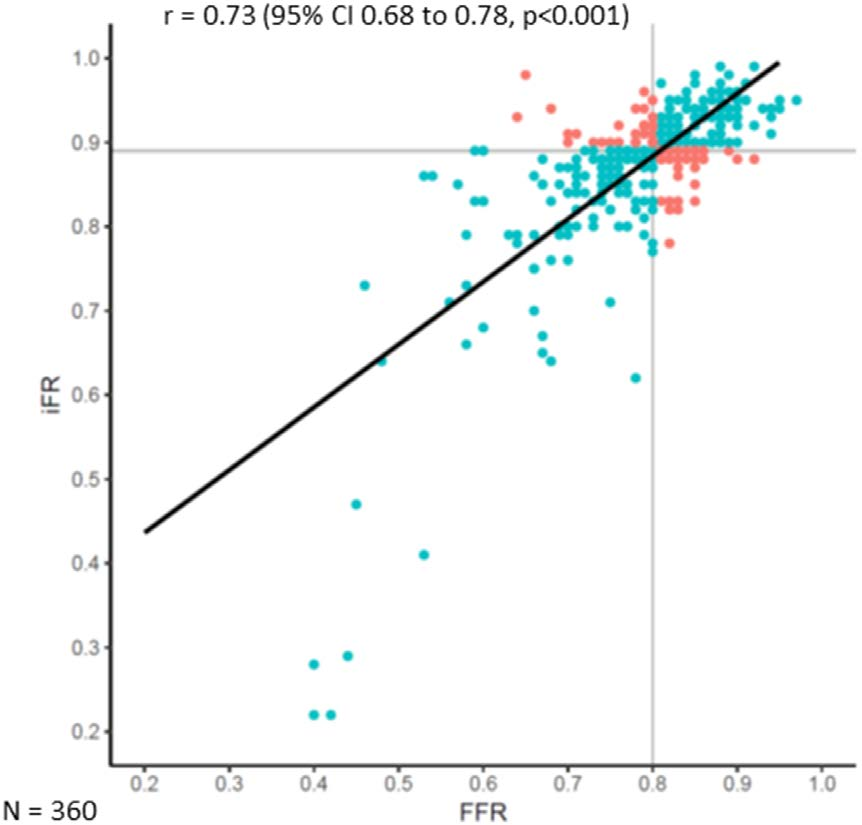
**Scatter plot showing the relationship between fractional flow reserve (FFR) and instantaneous wave-free ratio (iFR) values.** The black line represents the line of best fit. The gray lines represent the respective cutoff values for FFR (≤0.80) and iFR (≤0.89). Concordant cases are colored blue; discordant cases are colored orange.

A total of 360 coronary vessels (345 patients) were included for analysis from 6 centers (Imperial College London Hammersmith Hospital, St. Marianna University School of Medicine Yokohama City Seibu Hospital, Toda Central General Hospital, Kanazawa Cardiovascular Hospital, Okaya City Hospital, and Hospital Clínico San Carlos). The mean age was 64.4±10.3 years (76% men). The mean respective systolic and diastolic blood pressures on admission were 136±23 and 70±12 mm Hg, and mean heart rate was 70±12 bpm. The most frequently assessed vessel was the left anterior descending artery (81% [290 of 360]). Mean percentage diameter stenosis and lesion length were 51.6±13.5% and 20.9±14.8 mm, respectively. Full description of baseline, vessel, and stenosis characteristics are provided in Table 1. Median FFR and iFR values were 0.80 (interquartile range, 0.75–0.85) and 0.89 (interquartile range, 0.86–0.92), respectively. The relationship between FFR and iFR values is displayed in Figure 2 (R=0.73; *P*<0.001).

**Table 1.**
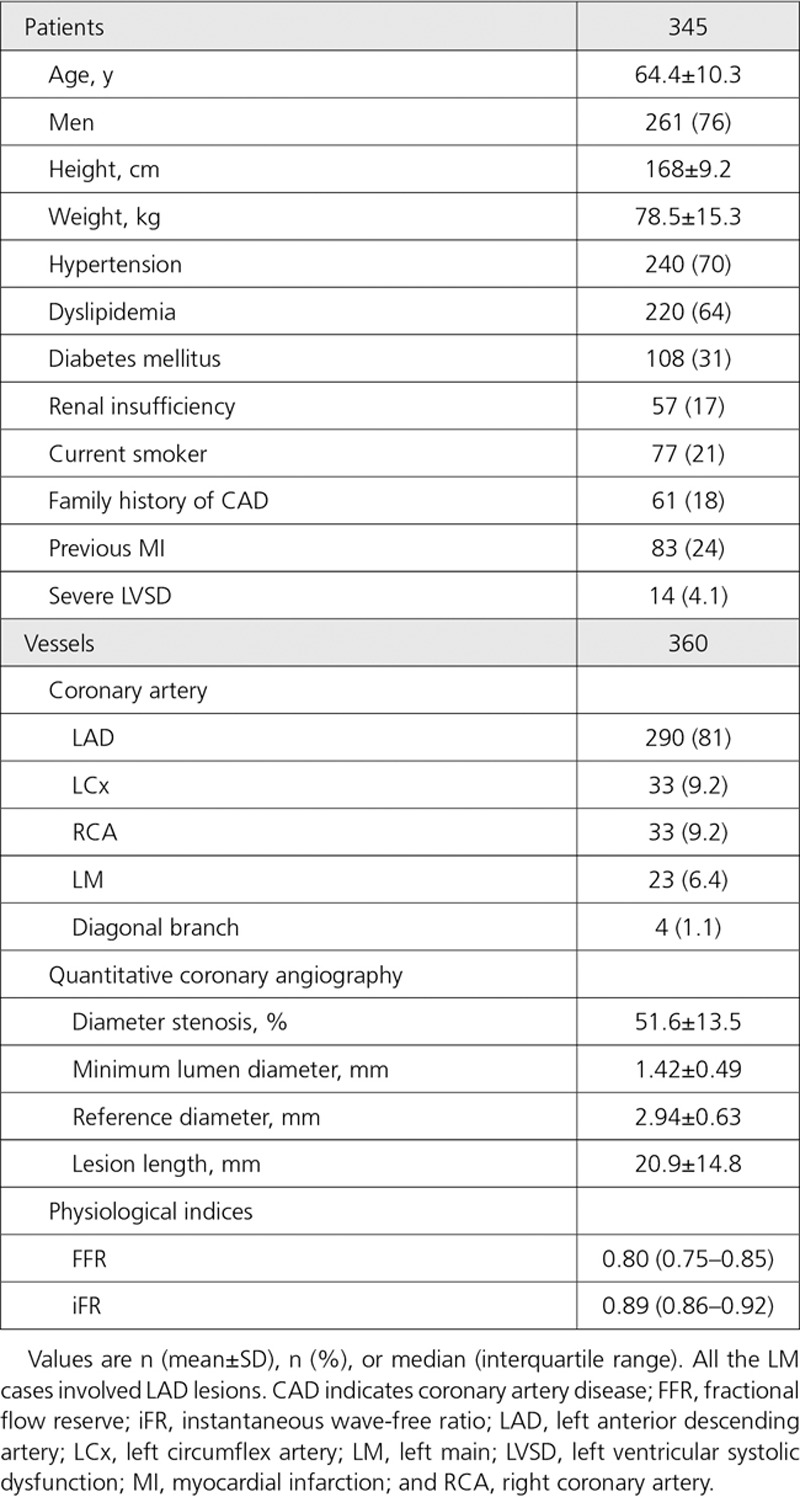
Patient and Vessel Characteristics

### Classification by FFR/iFR Values and iFR Pressure-Wire Pullback

FFR agreed with iFR in 78.1% (281 of 360) of cases, consisting of FFR+/iFR+ (n=154; 42.7%) and FFR−/iFR− (n=127; 35.3%). FFR disagreed with iFR in 21.9% (79 of 360) of cases, consisting of FFR−/iFR+ (n=38; 10.6%) and FFR+/iFR− (n=41; 11.4%). The iFR pullback curve, the physiological pattern of disease, was classified as 47.5% (171 of 360) predominantly physiologically focal and 52.5% (189 of 360) predominantly physiologically diffuse (Table 2).

**Table 2.**
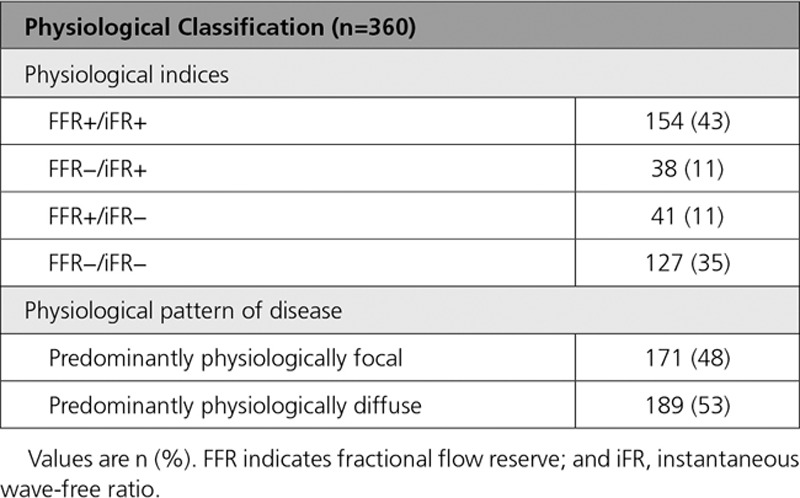
Classification by FFR/iFR and iFR Pressure-Wire Pullback

### Comparison Among Groups Classified by FFR and iFR

Clinical and lesion characteristics between concordant (FFR+/iFR+ and FFR−/iFR−) and discordant (FFR−/iFR+ and FFR+/iFR−) groups are displayed in Table I in the Data Supplement. There were no differences in patient and lesion characteristics between 2 groups. The prevalence of proximal lesions, defined as Syntax segments 1, 5, 6, and 11,^11^ was also similar (52.3% [147 of 281] versus 50.6% [40 of 79]; *P*=0.79). More specifically, stenosis location in the left main or ostial left anterior descending artery showed some tendency for FFR/iFR discordance; however, this did not meet statistical significance within the current dataset (9.6% [27 of 281] versus 17.7% [14 of 79]; *P*=0.071).

**Figure 3.**
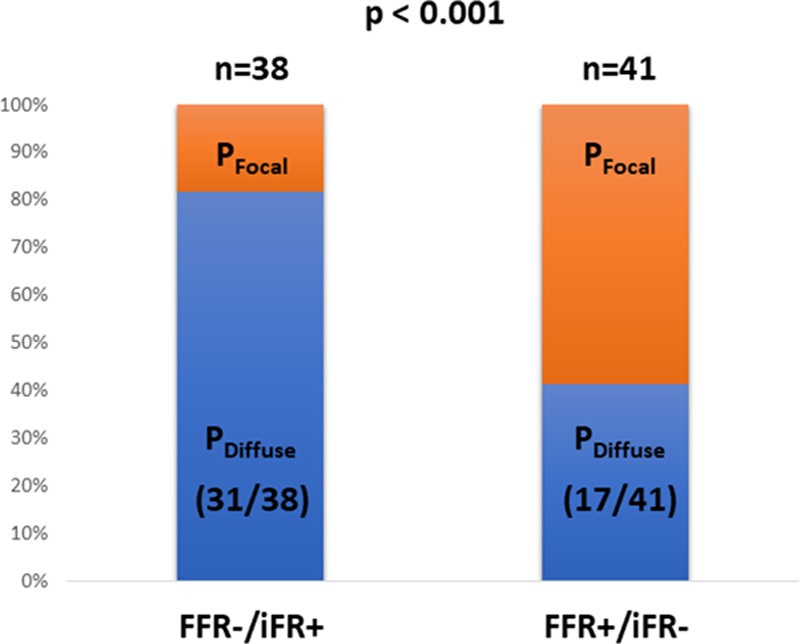
**The association between physiological pattern of disease and fractional flow reserve (FFR)/instantaneous wave-free ratio (iFR) discordance.** FFR−/iFR+ was significantly more associated with physiologically diffuse pattern of disease, whereas FFR+/iFR− was significantly more associated with physiologically focal pattern of disease. P_Diffuse_ indicates predominantly physiologically diffuse; and P_Focal_, predominantly physiologically focal.

In the FFR/iFR discordant (FFR−/iFR+ and FFR+/iFR−) groups, mean FFR and iFR values were 0.80±0.05 and 0.89±0.03, respectively. Baseline, vessel, and anatomic stenosis characteristics were all comparable between 2 discordant groups (*P*>0.05 for all; Table 3). Only the physiological pattern of disease as determined by the iFR pullback was significantly different between FFR−/iFR+ and FFR+/iFR− groups. In comparison to the FFR+/iFR− group, the FFR−/iFR+ group demonstrated a significantly higher prevalence of predominantly physiologically diffuse (82% [31 of 38] versus 42% [17 of 41]; *P*<0.001; Figure 3). Of note, within the discordant groups, there were no patients in whom coronary physiology was measured in multiple vessels. Accordingly, both patient and vessel number were the same (n=79) in the discordant groups.

**Table 3.**
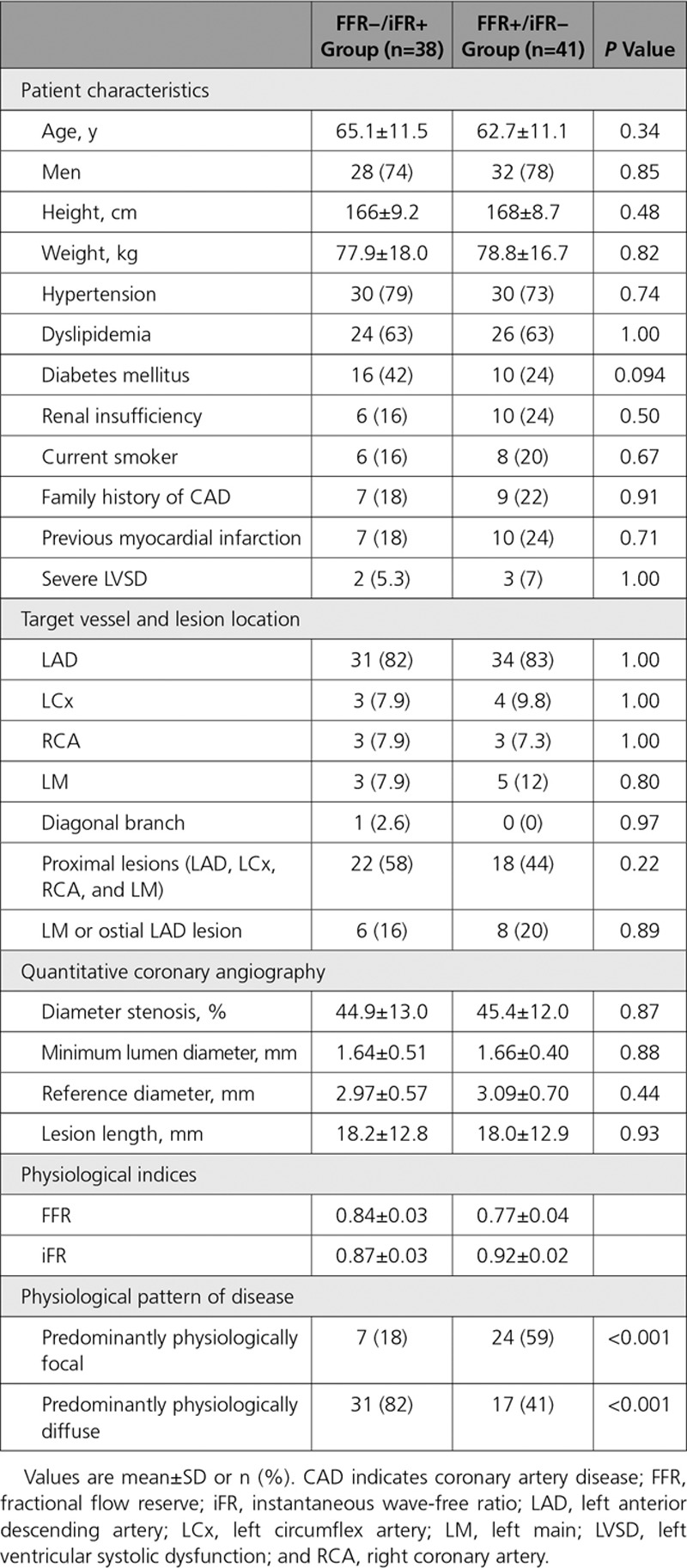
Comparison of 2 Discordant Groups

## Discussion

This study sought to determine whether the physiological pattern of coronary artery disease was an influencing factor for FFR/iFR discordance. The main findings of this study were as follows. First, unlike patient, vessel, or stenosis characteristics, the physiological pattern of disease was identified as a significant influencing factor for FFR/iFR discordance. Second, FFR+/iFR− discordance was significantly more associated with a physiologically focal pattern of disease. Last, FFR−/iFR+ discordance was significantly more associated with a physiologically diffuse pattern of disease.

### FFR/iFR Discordance—Influencing Clinical Features

It is well recognized that FFR and iFR disagree in the hemodynamic significance of a coronary lesion in ≈20% of cases.^4^ To date, several studies have investigated the clinically important topic of FFR/iFR discordance. Previous works have demonstrated that the discordance was observed more frequently in lesions in the proximal segment^5^ or left main and proximal left anterior descending artery lesion location^6^ compared with other lesion locations. Patient characteristics too have also been demonstrated to influence FFR/iFR discordance, with female sex and comorbidity with diabetes mellitus^7^ identified as significant influencing factors. The findings of the current study provided some consistency with these previous observations. Namely, most proximal stenosis location (left main/ostial left anterior descending artery) and patient comorbidity with diabetes mellitus demonstrated a tendency for determining FFR/iFR discordance (*P*=0.071 and *P*=0.094, respectively). In addition to corroborating these known factors, the current study identified that the physiological pattern of disease determined by pressure-wire pullback further influenced the discordance.

### FFR/iFR Discordance and the Physiological Pattern of Disease—Helping to Refine the Underlying Physiological Mechanism

The physiological mechanism underlying FFR/iFR discordance has recently been proposed as being due to differential coronary flow responses to pharmacological hyperemia.^8^ Specifically, FFR+/iFR− discordance was characterized by high hyperemic coronary flow velocity and FFR−/iFR+ discordance by low hyperemic coronary flow velocity. The findings of the present study may help provide a more refined mechanistic explanation for FFR/iFR discordance.

In the fluid dynamic equation that determines the degree of pressure loss across a stenosis because of variations in coronary flow velocity, linear and quadratic components are highly dependent on the geometry of the lesion itself. Although combined measurements of coronary pressure and flow were not performed in the current study, our findings suggested that in physiologically diffuse disease, frictional losses along the length of the vessel would be the predominant mode of a pressure energy loss, evident at rest (iFR+) and with only minimal increase during hyperemia (FFR−). Conversely, in physiologically focal disease, separation losses in the near vicinity of the stenosis itself would be the predominant mode of a pressure energy loss, minimally present at rest (iFR−) and evident only during hyperemia (FFR+).

### Clinical Implications

Discordance between FFR and iFR has been demonstrated to be multifactorial in origin, consisting of patient, anatomic, and physiological diversities.^5–8^ Such factors should be considered when interpreting the results of FFR/iFR discordant cases. However, given that the majority of discordance occurs close to the respective FFR and iFR cut points as in the literature,^5–8^ the clinical importance of FFR/iFR discordance itself should be interpreted with a degree of caution. In our datasets, the respective mean FFR and iFR values were also 0.80±0.05 and 0.89±0.03 in the discordant groups. Within this borderline diagnostic ranges, clinical outcome data are conflicting and only available from relatively small nonrandomized studies and are a subject of debate.^12–14^ More specifically, 1 study demonstrated the safety of deferral of percutaneous coronary intervention (PCI) in patients with gray-zone FFR (between 0.75 and 0.80) and suggested the prognostic threshold for FFR was in fact much lower (FFR, 0.67).^12^ A more recent study has also shown no significant difference in major adverse clinical events between PCI and medical therapy in the FFR gray-zone,^13^ whereas others have shown that PCI compared with medical therapy was associated with better clinical outcomes in patients with gray-zone FFR.^14^

Although there are no clinical outcome–related data to define the optimal treatment strategy when FFR/iFR discordance occurs, the predominance of physiologically focal or diffuse disease might be used to determine the treatment strategy if revascularization is contemplated. For example, in FFR+/iFR− discordance with an associated physiologically focal disease pattern, focal PCI may provide a preferred treatment strategy. However, if physiological values are borderline, it should be reconsidered whether the lesion causes sufficient ischemia to require stenting in patients with likely preserved coronary flow. Similarly, in FFR−/iFR+ discordance with an associated physiologically diffuse pattern, consideration should be given to the efficacy of PCI over long segments of disease. Even though resting physiology may support the indication of revascularization, clinicians should consider the balanced risks and benefits for revascularization with long stenting, which may not achieve an optimum post-PCI physiological result or good long-term outcomes.^15^

In summary, the routine performance of coronary pressure-wire pullback could better help clinicians determine the revascularization strategy in patients with intermediate-severity coronary artery disease, above and beyond simply the FFR or iFR value.

### Study Limitations

This study is not without limitation. First, owing to the retrospective registry-based design of this study, potential for selection bias of patients undergoing iFR pullback for clinical indications must be considered, and we could not provide information on baseline ratio of distal coronary pressure to aortic pressure during whole cardiac cycle. However, the value of such a registry-based approach reflects both real-world practice and clinically encountered disease populations. In addition, median FFR and iFR values in the present study were 0.80 and 0.89, respectively, whereas those in previous reports ranged 0.81 to 0.87 for FFR and 0.90 to 0.94 for iFR.^5–8^ Therefore, the populations in this study were comparable to those previously published in the literature.

Second, our findings should only be considered applicable to patients with isolated stable coronary artery disease. It has been demonstrated that acute coronary syndrome, cardiac hypertrophy, and severe aortic stenosis could attenuate the hyperemic response of the microcirculation to adenosine.^16^ Accordingly, it is likely the prevalence (and possibly mechanism) of FFR/iFR discordance may be different in these selected populations.

Third, in the present study, combined measurements of coronary flow velocity were not performed. Such measurements would be required to fully determine our proposed association between hyperemic coronary flow characteristics and physiological disease pattern, which remains speculative.

Finally, there is no clear criteria to define the physiological pattern of disease as assessed by coronary pressure-wire pullback. Although several studies proposed focal (abrupt) pattern and diffuse (gradual) pattern on FFR pullback, all definitions were different depending on investigators.^17,18^ Recent report demonstrated a simple predictive equation could accurately evaluate the component contributions of individual stenoses in serial lesions using FFR pullback, which might be helpful for determining the physiological pattern of disease in the future.^19^ As a result, currently, classification of the physiological pattern of disease is operator dependent and thus prone to both intra and interoperator variability. Mindful of this, within the present study, each iFR pullback trace was judged by the consensus opinion of 3 expert interventional cardiologists who reviewed the pullback data together. However, such an approach does not fully replicate clinical practice where data are often interpreted only by the performing physician. Further studies are being conducted to investigate the intraobserver and interobserver differences in the interpretation of pressure-wire pullback that may alter clinical decision-making.

### Conclusions

The physiological pattern of disease is an important influencing contributing factor for the discordance between FFR and iFR. Specifically, FFR+/iFR− discordance is more frequently associated with a focal disease pattern, and FFR−/iFR+ discordance is more frequently associated with a diffuse disease pattern.

## Acknowledgments

We appreciate all the staff of catheter laboratory at collaborating centers for their effort and understanding to clinical research work.

## Disclosures

Drs Warisawa and Davies report consultancy work for Philips. Dr Davies is an inventor of instantaneous wave-free ratio technology and holds intellectual property, which is under license. Drs Cook, Al Lamee, Sen, Petraco, and Nijjer have received speaker’s honoraria from Philips. The other authors report no conflicts.

## Supplementary Material
