# Supplementary material for: Physiological Pattern of Disease Assessed by Pressure-Wire Pullback Has an Influence on Fractional Flow Reserve/Instantaneous Wave-Free Ratio Discordance: Insights From the Multicenter AJIP Registry
Source: Circ Cardiovasc Interv. 2019 May 14;12(5):e007494. doi: 10.1161/CIRCINTERVENTIONS.118.007494 (PMC6553990; doi:10.1161/CIRCINTERVENTIONS.118.007494)
Supplement: Supplementary file 1 [file hcv-12-e007494-s001.pdf]

SUPPLEMENTAL MATERIAL

-Online Figure-

Online Figure 1. AJIP multicenter registry

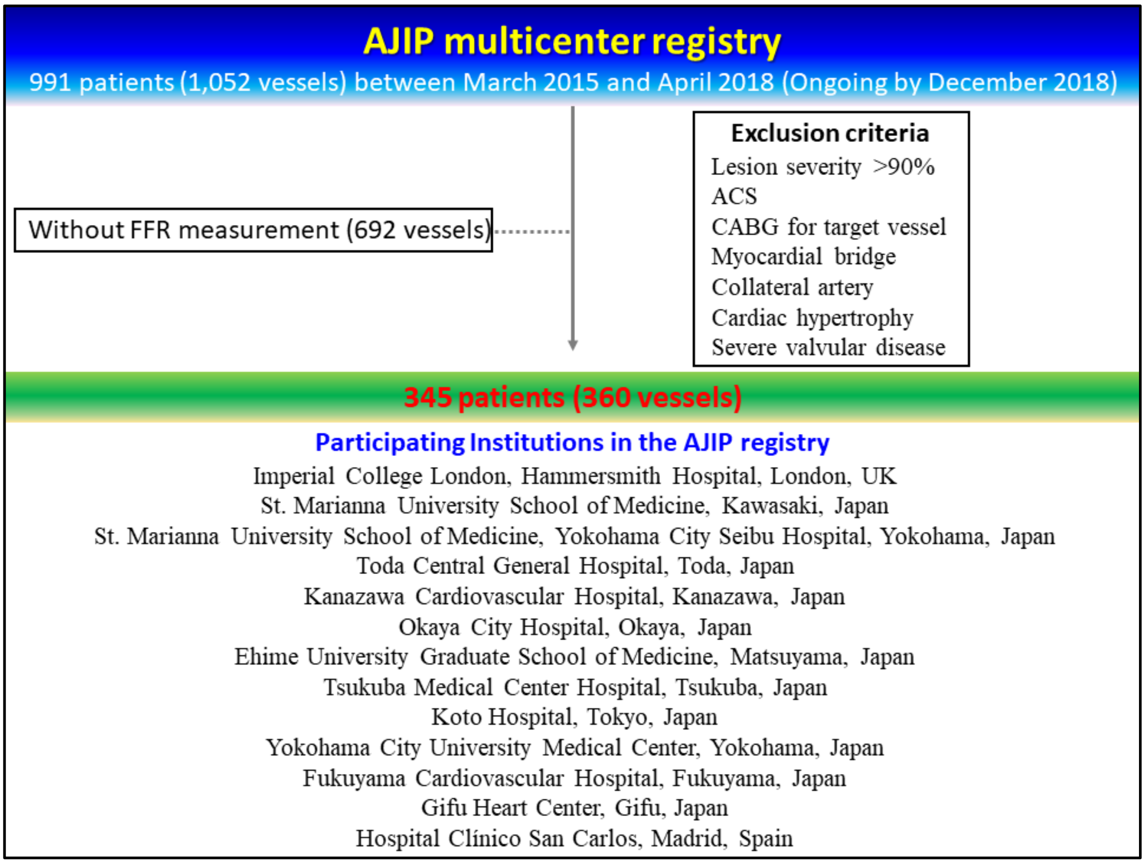

This registry includes 13 international centers in Europe and Japan and will cover the cases by December 2018 to investigate the utility and possibility of iFR pressure wire pullback. The 360 intermediate coronary lesions in 345 patients with combined measurement of iFR, iFR-pullback and FFR were analyzed in this study.

ACS: acute coronary syndrome; CABG: coronary artery bypass grafting; FFR: fractional flow reserve; iFR: instantaneous wave-free ratio

-Additional Table-

Supplemental Table 1. Comparison between concordant and discordant groups

	**Concordant Group (FFR+/iFR+, FFR-/iFR-, n=281)**	**Discordant Group (FFR-/iFR+, FFR+/iFR-, n=79)**	**p Value**
Patient characteristics			
Age, yrs	64.7 ± 9.9	63.8 ± 11.3	0.49
Male	212 (75)	60 (76)	1.00
Height, cm	168 ± 9.2	167 ± 8.9	0.22
Weight, kg	79.0 ± 14.8	78.4 ± 17.2	0.77
Hypertension	192 (68)	60 (76)	0.24
Dyslipidemia	178 (63)	50 (63)	1.00
Diabetes mellitus	88 (31)	26 (33)	0.89
Renal insufficiency	45 (16)	16 (20)	0.47
Current smoker	69 (25)	14 (18)	0.20
Family history of CAD	48 (17)	16 (20)	0.63
Previous MI	69 (25)	17 (22)	0.68
Severe LVSD	11 (3.9)	5 (6.3)	0.54
Target vessel and lesion location			
LAD	225 (80)	65 (82)	0.78

LCx	26 (9.2)	7 (8.9)	1.00
RCA	27 (9.6)	6 (7.6)	0.74
LM	15 (5.3)	8 (10)	0.20
Diagonal branch	3 (1.1)	1 (1.3)	1.00
Proximal lesion (LAD, LCx, RCA and LM)	147 (52)	40 (51)	0.79
LM or ostial LAD lesion	27 (9.6)	14 (18)	0.071

Values are n, mean ± SD, n (%). CAD = coronary artery disease; FFR: fractional flow reserve; iFR: instantaneous wave-free ratio; LAD = left anterior descending artery; LCx = left circumflex artery; LM = left main; LVSD = left ventricular systolic dysfunction; MI = myocardial infarction; RCA = right coronary artery.
